# Development and validation of a sensitive liquid chromatography tandem mass spectrometry assay for the simultaneous determination of ten kinase inhibitors in human serum and plasma

**DOI:** 10.1007/s00216-020-03031-7

**Published:** 2020-11-06

**Authors:** Fatemeh Aghai, Sebastian Zimmermann, Max Kurlbaum, Pius Jung, Theo Pelzer, Hartwig Klinker, Nora Isberner, Oliver Scherf-Clavel

**Affiliations:** 1 Department of Internal Medicine II, University of Würzburg Medical Center, Oberdürrbacher Str. 6, 97080 Würzburg, Germany; 2 Institute of Pharmacy and Food Chemistry, University of Würzburg, Am Hubland, 97074 Würzburg, Germany; 3 Department of Internal Medicine I, University of Würzburg Medical Center, Oberdürrbacher Str. 6, 97080 Würzburg, Germany; 4 Core Unit Clinical Mass Spectrometry, University of Würzburg Medical Center, Oberdürrbacher Str. 6, 97080 Würzburg, Germany; 5 Department of Internal Medicine I, Division of Pneumonology, University of Würzburg Medical Center, Oberdürrbacher Str. 6, 97080 Würzburg, Germany

**Keywords:** Kinase inhibitors, Therapeutic drug monitoring, Liquid chromatography tandem mass spectrometry (LC-MS/MS), Afatinib, Osimertinib

## Abstract

A liquid chromatography tandem mass spectrometry method for the analysis of ten kinase inhibitors (afatinib, axitinib, bosutinib, cabozantinib, dabrafenib, lenvatinib, nilotinib, osimertinib, ruxolitinib, and trametinib) in human serum and plasma for the application in daily clinical routine has been developed and validated according to the US Food and Drug Administration and European Medicines Agency validation guidelines for bioanalytical methods. After protein precipitation of plasma samples with acetonitrile, chromatographic separation was performed at ambient temperature using a Waters XBridge® Phenyl 3.5 μm (2.1 × 50 mm) column. The mobile phases consisted of water-methanol (9:1, v/v) with 10 mM ammonium bicarbonate as phase A and methanol-water (9:1, v/v) with 10 mM ammonium bicarbonate as phase B. Gradient elution was applied at a flow rate of 400 μL/min. Analytes were detected and quantified using multiple reaction monitoring in electrospray ionization positive mode. Stable isotopically labeled compounds of each kinase inhibitor were used as internal standards. The acquisition time was 7.0 min per run. All analytes and internal standards eluted within 3.0 min. The calibration curves were linear over the range of 2–500 ng/mL for afatinib, axitinib, bosutinib, lenvatinib, ruxolitinib, and trametinib, and 6–1500 ng/mL for cabozantinib, dabrafenib, nilotinib, and osimertinib (coefficients of correlation ≥ 0.99). Validation assays for accuracy and precision, matrix effect, recovery, carryover, and stability were appropriate according to regulatory agencies. The rapid and sensitive assay ensures high throughput and was successfully applied to monitor concentrations of kinase inhibitors in patients.

**Supplementary Information:**

The online version contains supplementary material available at 10.1007/s00216-020-03031-7.

**Figure:**
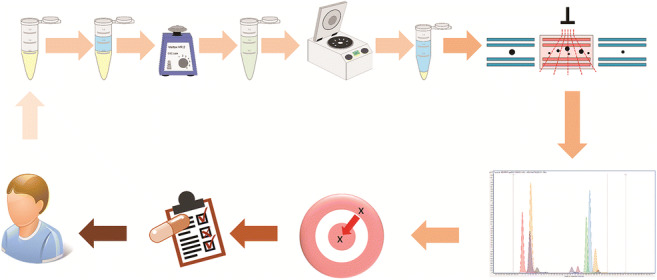
Graphical abstract

## Introduction

Almost 20 years after the approval of imatinib, oral anticancer therapy has become an integral part in the treatment regimens of various malignancies [1] and other diseases such as graft versus host disease (GvHD) [2] and even COVID-19 [3]. One group of oral anticancer drugs comprises so-called kinase inhibitors (KI) such as osimertinib and afatinib which share a similar mechanism of action by inhibiting protein kinases in malignant cells. Protein kinases play a key role in cellular signaling pathways regulating cell proliferation and differentiation or programmed cell death. Their effect is mediated by catalyzing the phosphorylation of protein residues (e.g., tyrosine residues). Genetic alterations may lead to improper regulation, promoting the development of cancer or other diseases [4].

Osimertinib for instance is an epidermal growth factor receptor (EGFR) tyrosine kinase inhibitor that binds to certain mutant forms of EGFR that predominate in non-small cell lung cancer (NSCLC) tumors [5]. Afatinib as another example is a selective and irreversible ErbB family blocker such as EGFR and the human epidermal growth factor receptor 2 and is approved for the treatment of locally advanced or metastatic NSCLC [6].

Considering the complex pharmacokinetics (PK) of KI, a high level of interindividual variability in drug exposure due to varying absorption, distribution, metabolism, and excretion has been demonstrated for many KI [7]. As KI are administered orally, drug formulation, food intake, stomach pH, solubility, disturbed gastrointestinal adsorption, or first-pass hepatic metabolism may lead to variability in oral bioavailability. Moreover, almost all KI are predominantly metabolized by isoenzymes of the cytochrome P450 family (CYP) [8–11]. Variability in expression and activity of CYP enzymes contribute to interindividual differences in drug metabolism and drug clearance [12, 13]. This effect is particularly enhanced by concomitant administration of CYP inducers or inhibitors and thus common in patients with polypharmacotherapy such as cancer and GvHD patients [14–16]. In addition, polymorphisms in genes encoding drug transporters (e.g., ABC transporter) involved in the absorption and excretion of many KI influence influx and efflux processes and subsequently drug distribution and exposure [17, 18].

A targeted mechanism of action must not be confused with a total lack of side effects. KI therapy comes along with a wide range of mild to severe adverse events (AE), often of the augmented type, which may be enhanced by drug overexposure [19]. Intolerable AE and toxicity frequently lead to dose reductions or treatment discontinuations endangering treatment efficacy [20, 21]. On the other hand, tumors progress within months after initially responding to targeted therapy in many cases. At least in some cases, this behavior might be driven by subtherapeutic concentrations, triggering the development of resistance mechanisms [22, 23]. In contrast to intravenous chemotherapy, non-adherence might be an additional factor leading to varying drug exposure.

For many KI, large differences in plasma concentrations between patients have been described [24–28]. One strategy to prevent under- or overexposure of drug concentrations and monitor adherence is therapeutic drug monitoring (TDM). For some agents, TDM has already proven to be feasible and useful [29]. Strong evidence exists for imatinib in chronic myelogenous leukemia [30, 31]. For alectinib [32], axitinib, crizotinib [32], trametinib [26], and vemurafenib [33–36], a PK target associated with either efficacy or toxicity has been established but has not been evaluated in clinical studies yet [37]. However, for most kinase inhibitors, no information about the potential benefit of TDM is available, as exposure-response relationships and consequently PK targets have not been established.

To further investigate concentration-dependent effects and the potential use of TDM for KI in daily clinical routine, the aim of this work was the development and validation of a simple and time-saving liquid chromatography tandem mass spectrometry (LC-MS/MS) method for the quantification of an analyte panel comprising the KI afatinib (AFA), axitinib (AXI), bosutinib (BOS), cabozantinib (CAB), dabrafenib (DAB), lenvatinib (LEN), nilotinib (NIL), osimertinib (OSI), ruxolitinib (RUX), and trametinib (TRA). The assay was so far successfully applied to analyze serum levels of one patient taking AFA and two patients taking OSI.

## Material and methods

### Chemicals

Axitinib and lenvatinib were purchased from Alsachim (Illkirch-Graffenstaden, France), and afatinib, bosutinib monohydrate, cabozantinib malate, dabrafenib mesylate, osimertinib mesylate, and ruxolitinib were acquired from Carbosynth Ltd. (Berkshire, UK). Nilotinib was bought from LC Laboratories (Woburn, MA, USA) and trametinib from Ark Pharm (Arlington Heights, IL, USA). ^2^H_6_-Afatinib, ^2^H_9_-bosutinib, ^2^H_9_-dabrafenib, ^2^H_5_-lenvatinib, ^13^C^2^H_3_-osimertinib, and ^13^C^2^H_6_-trametinib were obtained from Alsachim (Illkirch-Graffenstaden, France), and ^13^C^2^H_3_-axitnib, ^2^H_4_-cabozantinib, ^2^H_6_-nilotinib, and ^2^H_4_-ruxolitinib were purchased from Biozol Diagnostica GmbH (Eching, Germany). Ammonium bicarbonate was bought from Sigma-Aldrich Inc. (Steinheim, Germany). Acetonitrile, methanol, water (all LC-MS-grade), and dimethyl sulfoxide were purchased from Merck KGaA (Darmstadt, Germany). Blank human plasma was provided by the Institute for Transfusion Medicine and Haemotherapy of the University of Wuerzburg Medical Center.

### Stock solutions and working solutions

Stock solutions of all analytes were separately prepared at a concentration of 1 mg/mL (calculated as free base) in dimethyl sulfoxide (DMSO). Two independent stock solutions were used for the preparation of calibration standards and quality control (QC) samples. The stock solutions were combined and diluted with methanol to obtain working solutions. Stock solutions for all internal standards (IS) were separately prepared at a concentration of 1 mg/mL in DMSO and were diluted with DMSO to prepare an IS working stock solution at a concentration of 20 μg/mL. Subsequently, the mixture was diluted with acetonitrile (ACN) to yield the precipitating agent at an IS concentration of 50 ng/mL (for each IS). Stock solutions were stored at − 80 °C in 2.0 mL polypropylene tubes and working solutions were stored at − 20 °C in glass tubes (0.5 mL). AXI and DAB were stored in amber-colored tubes (2.0 mL) due to their light sensitivity [38, 39].

### Calibration standards and quality control samples

Nine hundred fifty microliters of blank human plasma was spiked with 50 μL calibration standard working solution to obtain the highest calibration standard, containing all KI but OSI. The highest QC sample was established by spiking 50 μL of the separately prepared working solution to 950 μL of blank human plasma. QC low (QC-L), QC middle (QC-M), QC high (QC-H), and the remaining target calibrators were obtained by serial dilution with human plasma.

To determine the lower limit of quantification (LLOQ), QC-LLOQ was prepared equivalently. Aliquots of 50 μL for each concentration level were stored at − 20 °C.

Both validation and analytical runs contained nine calibration levels over the range (one replicate per level). All validation runs, except for stability assessment runs, consisted of five replicates of each QC level (in total *n* = 20 per run). Stability assessment runs and analytical runs included QC-H, QC-M, and QC-L in triplicates (in total *n* = 9 per run).

OSI calibration standards and QC samples were first prepared in methanol in a 20-fold higher concentration compared to the final concentrations in human plasma. Nine hundred fifty microliters of blank human plasma was then spiked with 50 μL of each concentration level to obtain final calibrators and QC samples.

The calibration range for CAB, DAB, NIL, and OSI was 6–1500 ng/mL. The concentrations for QC-LLOQ, QC-L, QC-M, and QC-H were 6, 15, 600, and 1200 ng/mL, respectively.

AFA, AXI, BOS, LEN, RUX, and TRA calibration was applied in the range of 2–500 ng/mL. QC samples for these analytes were prepared at concentrations of 2, 5, 200, and 400 ng/mL for QC-LLOQ, QC-L, QC-M, and QC-H, respectively. Dilution of QC-H yielded QC-M (1:2), which was used to prepare an intermediate (level 1) (1:4). Another intermediate (level 2) was prepared from this solution (1:3 for CAB, DAB, NIL OSI, or 2:5 for AFA, AXI, BOS, LEN, RUX, TRA, respectively). Dilution of intermediate level 2 was used to prepare QC-L (1:4) which then again was diluted to QC-LLOQ (2:5).

### Collection of patient samples

Immediately after sample collection by venipuncture, patient blood samples were centrifuged for 10 min at 4500 rcf at 18 °C. Serum was subsequently isolated and aliquots of 300 μL were stored at − 80 °C. Light protection of AXI samples was implemented by packaging samples in opaque plastic bags for transportation. Samples were thawed at room temperature prior to processing while being protected from daylight. OSI samples were thawed in the fridge (8 °C).

### Sample preparation

For each validation and analytical run, freshly prepared QC samples and calibrators were used. Samples were prepared by protein precipitation with ACN as precipitation agent. One hundred fifty microliters of ice-cooled ACN containing IS was added to 50 μL sample, calibrator, or QC sample. The samples were vortexed for 15 s and centrifuged for 5 min at 4 °C and 11,000 rcf. Fifty microliters of supernatant was subsequently transferred to another tube containing 450 μL mobile phase A and was vortexed for an additional 10 s. Three hundred microliters was transferred to an autosampler vial with polypropylene insert. Forty microliters was injected into the chromatographic system.

### Chromatographic equipment and conditions

Chromatographic separation was carried out using an Agilent 1290 Infinity LC System equipped with binary pump, autosampler, and thermostatted column compartment (Waldbronn, Germany). Chromatographic separation was achieved using a Waters XBridge® Phenyl 3.5 μm (2.1 × 50 mm) column and an eluent consisting of water-methanol (9:1, v/v) with 10 mM ammonium bicarbonate as phase A and methanol-water (9:1, v/v) containing 10 mM ammonium bicarbonate as phase B. To degas mobile phases and for further purification, vacuum filtration with a Millipore filtering system consisting of a ground joint flask, glass funnel, glass frit base, and clamp (Millipore Corporation, Billerica, MA, USA) using Sartorius™ SARTOLON polyamide membrane filter, 0.45 μm (Sartorius Stedim Biotech GmbH, Goettingen, Germany), was used.

Gradient elution was applied at a flow rate of 400 μL/min using the following time program: 0–0.5 min 60% B, 0.5–2.00 min linear increase to 80% B, 2.00–5.00 min 80% B, 5.00–5.25 min linear decline to 60% B and remained at 60% B for the last 2 min. ACN-water (9:1, v/v) was used as needle wash solution. The temperature of the autosampler was kept at 10 °C. The column temperature was set to ambient temperature (controlled at 18 °C by air conditioning). Acquisition time was 7.0 min per run.

### Mass spectrometer and conditions

For detection, a Sciex QTRAP 4500 MD mass spectrometer (Framingham, MA, USA) meeting the legal requirements for medical devices (MD) was used. Pneumatically assisted electrospray ionization (ESI) in positive mode and multiple reaction monitoring (MRM) were configured for ionization and fragmentation, respectively. The linear ion trap was not used in this analysis. Source parameters were set as follows: ion spray gas 3000 Volt, nebulizer gas 30 AU, collision gas medium, curtain gas 30 AU, temperature 400 °C, heater gas 30 AU. The mass spectrometer settings for each compound are illustrated in Electronic Supplementary Material (ESM) Table S1.

### Data processing and illustrations

Analytical results were recorded and processed using Analyst Software 1.6.3 MD (Sciex, Framingham, MA, USA). Validation results were processed using Microsoft Excel Version 13.36 (Microsoft Corporation, Redmond, WA, USA). Patient data were analyzed using R Version 3.6.0 (R Foundation, Austria). Plots were generated using the “ggplot2” package in R. Chemical structures were illustrated with ChemDraw Version 19.0.1.332 (PerkinElmer Informatics, Waltham, MA, USA).

### Method characterization

The assay was validated according to the European Medical Agency (EMA) (2011) and US Food and Drug Administration (2018) regulatory guidelines on bioanalytical method validation [40, 41].

#### Method robustness

The method’s robustness was monitored daily by comparing the intensity of the analytes’ and IS’ signal for each concentration and compound. Retention times of each analyte were compared to previous runs. The system’s pressure during analysis and prior to analysis, variations in pressure, and its possible sources were also monitored. Another lot of column and solvents were used to check for differences in intensity and retention times. The pressure during a run was also randomly compared between samples and to previous runs for the same concentration.

To ensure the accuracy of freshly prepared calibrators and QC samples, they were measured against previous calibrators or QC samples. Robustness of the method was also evaluated by the guidelines’ requirements of intra-day and inter-day accuracy and precision. Imprecision was calculated to monitor daily variations and was not expected to be < 15%.

#### Calibration curves, linearity, and sensitivity

The ratio of analyte peak area and isotopically labeled IS was defined as response and the concentration as the independent variable. The upper limit of quantitation (ULOQ) was defined as the concentration of the highest calibration level with accuracy between 85 and 115% (*n* = 5) and a precision within 15%. LLOQ was defined as the concentration of the lowest calibrator with an accuracy between 80 and 120% (*n* = 5) and a precision (expressed as coefficient of variance (CV) in percentage) within 20%. To obtain sensitivity, the response of the analyte at LLOQ was compared to the response obtained by the zero calibrator (matrix blank prepared with IS) and was expected to be at least 5 times higher (acceptance factor ≤ 1):

1 $$ \mathrm{Acceptance}\ \mathrm{Factor}=\frac{\mathrm{Peak}\ \mathrm{Area}\ \left(\mathrm{analyte}\right)\ \mathrm{at}\ \mathrm{zero}\ \mathrm{calibrator}\times 5}{\mathrm{Peak}\ \mathrm{Area}\ \left(\mathrm{analyte}\right)\ \mathrm{LLOQ}} $$

In addition, signal-to-noise (S/N) ratios were calculated by the Analyst Software for the lowest calibration level for each analyte on nine different days (= nine calibration runs). A 10:1 ratio was considered to be appropriate.

#### Selectivity

To differentiate the analytes and the IS from endogenous and other components in the matrix, selectivity was demonstrated by analyzing blank plasma samples from six different healthy donors. The impact of hemolytic, icteric, and lipemic serum was also tested as part of the selectivity assessment and matrix effect. Spiked QC samples in serum as matrix and blank serum were also measured to ensure the absence of interference in serum and matrix effects of serum.

All MRM traces were monitored and investigated for the absence of interference by matrix or crosstalk between MRM transitions.

Selectivity of the method was accepted if the response of blank samples was free of interference at the retention times of analytes and IS. The absence of interfering peaks was characterized by blank responses < 5% for IS and < 20% of LLOQ, respectively.

#### Carryover

Carryover was investigated by analyzing blank samples (prepared without IS) injected directly after ULOQ samples (1500 ng/mL or 500 ng/mL, *n* = 5). A mean signal ≤ 20% of LLOQ and less than 5% for IS was defined as absence of carryover effects. Five replicates were measured. Peak area was used for calculation.

#### Recovery and matrix effects

To investigate matrix effects, KI-free plasma from six healthy donors were used to prepare QC-H and QC-L samples (*n* = 6 per level). These samples in biological matrix were compared to QC-H and QC-L samples in ACN as matrix. Both batches were prepared by serial dilution as described in the “Calibration standards and quality control samples.” For each analyte and IS, matrix factor (MF) was calculated.

2 $$ \mathrm{MF}=\frac{\ \mathrm{Peak}\ \mathrm{Area}\ \mathrm{in}\ \mathrm{the}\ \mathrm{presence}\ \mathrm{of}\ \mathrm{matrix}\ }{\mathrm{Peak}\ \mathrm{Area}\ \mathrm{in}\ \mathrm{absence}\ \mathrm{of}\ \mathrm{matrix}} $$

In addition, the IS-normalized MF was calculated by dividing the MF of analyte by the MF of IS [40]. Matrix effects were considered absent if the CV of the IS-normalized MF calculated from the six lots of matrix was ≤ 15%.

To confirm efficient recovery, analytical results of blank KI-free plasma from six different healthy donors spiked with analyte after extraction (A.E.) (representing 100% recovery) were compared to samples from six different healthy donors spiked with analyte before extraction (B.E.) at QC-H, QC-M, and QC-L (*n* = 6 per level). The following equation was used to calculate recovery:

3 $$ \mathrm{Recovery}\%=\frac{\raisebox{1ex}{$\mathrm{Peak}\ \mathrm{Area}\ \left(\mathrm{analyte}\right)\mathrm{B}.\mathrm{E}.$}\!\left/ \!\raisebox{-1ex}{$\mathrm{Peak}\ \mathrm{Area}\ \left(\mathrm{internal}\ \mathrm{standard}\right)\mathrm{B}.\mathrm{E}.$}\right.}{\raisebox{1ex}{$\mathrm{Peak}\ \mathrm{Area}\ \left(\mathrm{analyte}\right)\mathrm{A}.\mathrm{E}.$}\!\left/ \!\raisebox{-1ex}{$\mathrm{Peak}\ \mathrm{Area}\ \left(\mathrm{internal}\ \mathrm{standard}\right)\mathrm{A}.\mathrm{E}.$}\right.}\times 100 $$

#### Dilution integrity

In case patient samples have a concentration higher than ULOQ, dilution integrity was tested by preparing samples above ULOQ (3000 ng/mL and 1000 ng/mL, respectively) using blank human plasma or physiological saline solution as diluents (1:2). Six replicates were measured.

#### Accuracy and precision

Accuracy and precision were evaluated using QC-H, QC-M, QC-L, and QC-LLOQ. Intra-day accuracy and precision were determined by measuring five replicates of every QC in a single run. Inter-day accuracy and precision were assessed by performing single runs with five replicates of each QC on three different days. The limits for accuracy and precision were ± 15% and CV ≤ 15%, respectively. According to guidelines, the limits for accuracy and precision (CV) of LLOQ were ± 20% and ≤ 20%, respectively. Total within-day and between-day imprecisions were calculated according to the method of Krouwer et al. [42].

4 $$ \mathrm{Precision}\ \left(\%\right)=\frac{\mathrm{standard}\ \mathrm{deviation}\ \left(\mathrm{SD}\right)\ \mathrm{per}\ \mathrm{QC}\ \mathrm{level}\ }{\mathrm{mean}\ \mathrm{concentration}\ \mathrm{per}\ \mathrm{QC}\ \mathrm{Level}} \times 100 $$

#### Stability of samples and stock solutions

Pre-preparative stability (bench-top stability) of KI in human plasma was investigated by comparing freshly prepared QC samples (QC-H and QC-L) to unprocessed QC samples kept at room temperature for 24 h without daylight and at room temperature for 48 h with or without daylight, in the fridge at 4 °C for 6 weeks and after incubation at 56 °C for 1 h. Post-preparative stability was tested by measuring processed samples after being kept in the autosampler for 24 h (autosampler stability).

Long-term stability of samples was tested by comparing freshly prepared QC in plasma to QC samples in plasma stored at − 20 °C or − 80 °C for 3 months. Freeze-thaw stability was investigated by measuring QC samples after three cycles of freezing at − 20 °C and thawing at ambient temperature for 2 h. Stability for OSI at different storage conditions and long-term stability were tested separately since preparation of calibration standards and QC samples for OSI had to be changed prior to validation.

Long-term stability of stock solutions in DMSO was tested by comparing freshly prepared solutions in DMSO with 4-month-old solutions kept at − 80 °C. Stability of working solutions in methanol was tested by comparing a freshly prepared working solution with a 4-month-old working solution which underwent several freeze (− 20 °C) and thaw cycles.

Stability was accepted, if the measured concentrations met the nominal concentration ± 15%.

#### Incurred sample reanalysis (ISR)

The accuracy of incurred samples was evaluated by reanalysis of study samples in separate runs at different days. About 10% of the samples from each run were analyzed again. The following equation was used for calculation according to EMA guidelines:

5 $$ \%\mathrm{difference}=\frac{\left(\mathrm{repeat}\ \mathrm{value}\hbox{--} \mathrm{initial}\ \mathrm{value}\right)\ }{\mathrm{mean}\ \mathrm{value}}\times 100 $$

### Clinical application

The method was applied to determine AFA and OSI concentrations in daily clinical routine.

One sample per visit was collected from adult patients receiving AFA and OSI for the treatment of EGFR-mutant metastatic lung adenocarcinoma who had a predicted prognosis of > 2 months. Data regarding the patients’ additional condition and co-medication were accessed by questionnaires and documented history (ESM Table S7).

Twenty-eight steady-state serum samples of three patients receiving AFA 20 mg (*n* = 3) or OSI 80 mg (*n* = 25) on a once daily schedule were collected during routine patient visits in an outpatient setting. Steady state was assumed 8 days after initiation of AFA and 15 days after initiation of OSI therapy according to the information obtained from the summary of product characteristics of Giotrif and Tagrisso, respectively [5, 6]. For both compounds, trough and non-trough levels were analyzed. The study was approved by the Ethics Commission of the University of Wuerzburg (ref199/18-am) and was conducted in accordance with the declaration of Helsinki. All patients gave written informed consent.

## Results

### Method characterization

#### Method robustness

The system’s pressure prior to analysis and during analysis was reproducible on a daily basis and compared to prior runs. Using a differing lot of column and solvents showed neither significant changes in the intensity of signals for the same concentration nor in the retention times of the analytes.

Freshly prepared calibrators and QC samples were first measured against an older set of calibrator and QC samples and were only used for validation or analytical runs if their accuracy met the accuracy and precision criteria of ± 15% and ≤ 15%, respectively. Hence, reproducibility of the method was guaranteed.

Finally, results of the inter-day and intra-day accuracy and precision according to the abovementioned validation guidelines (2.9.7) ensured the method’s robustness on a daily basis.

#### Calibration curves, linearity, and sensitivity

The mathematical model which fitted best was a weighted ($\frac{1}{conc^2}$) linear regression model for all analytes. The calibration curves were linear (*R*^2^ ≥ 0.995–0.999) and reproducible for all analytes. Calculation of the acceptance factor for each KI showed the method’s sensitivity at LLOQ (ESM Table S2). In addition, S/N ratios were calculated for the lowest calibration level and met the acceptance criteria 10:1 (ESM Table S2).

#### Selectivity

**Fig. 1:**
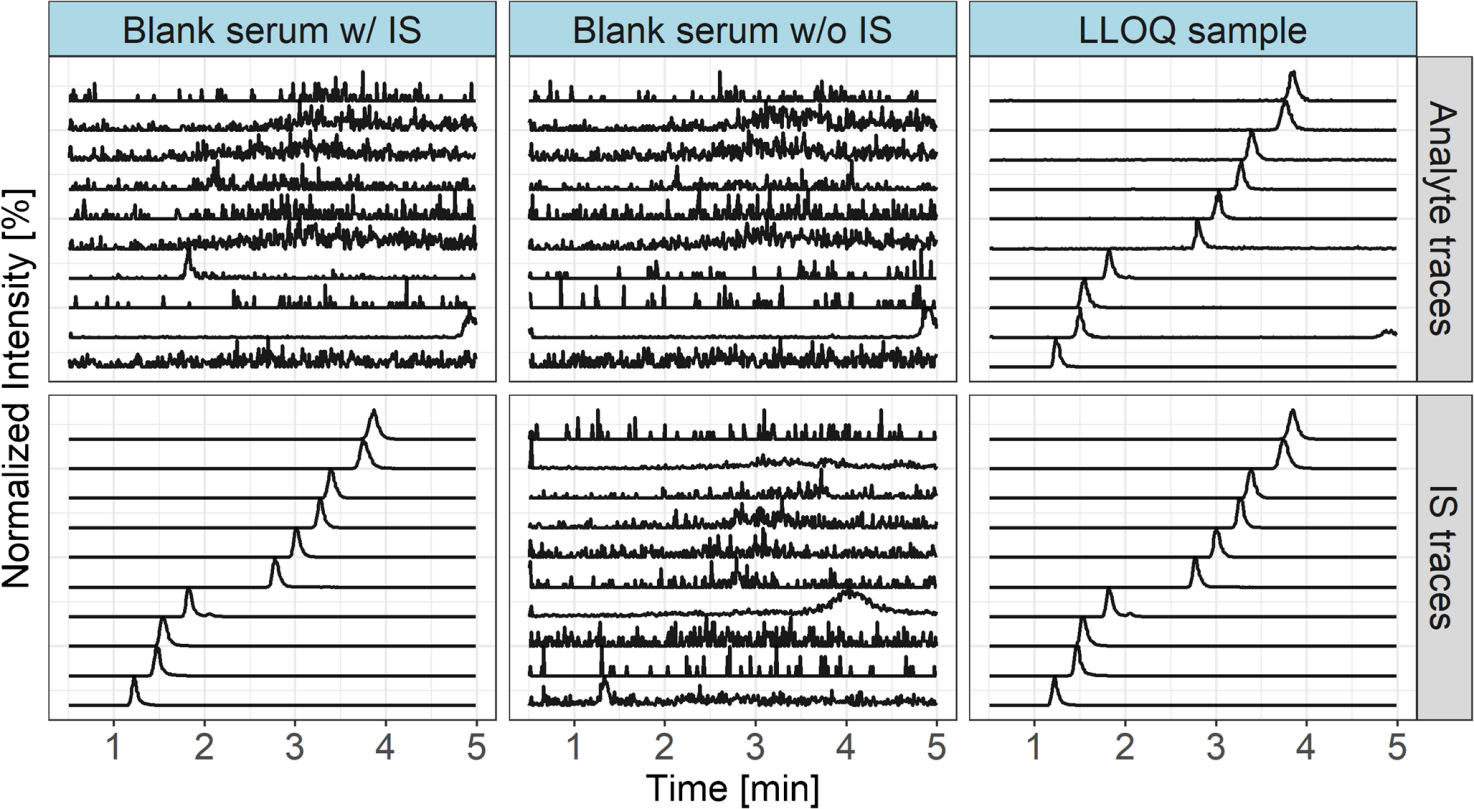
Overlaid MRM chromatograms of a blank sample without (w/o) and with (w/) IS and of an LLOQ sample stratified by MRM traces for the target analytes and the corresponding IS. Traces correspond to (from bottom to top): RUX/^2^H_9_-RUX, DAB/^2^H_9_-DAB, LEN/^2^H_5_-LEN, AXI/^13^C^2^H_3_-AXI, BOS/^2^H_9_-BOS, AFA/^2^H_6_-AFA, NIL/^2^H_6_-NIL, CAB/^2^H_4_-CAB, OSI/^13^C^2^H_3_-OSI, TRA/^13^C_6_-TRA

The response in the blanks were less than 20% of LLOQ and ≤ 5% for IS (ESM Table S2). When injecting IS of AXI, a small degree of crosstalk or contamination was detected in the MRM trace of the analyte at the retention time for AXI (Fig. 1 and ESM Figs. S1–S3). However, it did not exceed the required limit of 20% of LLOQ.

#### Carryover

Carryover of the analytes did not exceed 20% of LLOQ and carryover of IS were below 5% (ESM Table S2).

#### Recovery and matrix effects

Recovery results were reproducible and consistent for concentrations near the lower (91.1–110%) and upper limits of quantification (89.5–105%).

Hemolytic, icteric, and lipemic serum did not have any impact on accuracy and precision (ESM Table S3). Values were in the range of ± 15% (accuracy) and ≤ 15% (precision). Neither accuracy nor precision were affected when using serum as matrix instead of plasma (ESM Table S4).

The CV of the IS-normalized MF calculated for the 6 lots of matrix was less than 15% for all QC levels (range 2.86–10.1%). IS-normalized MF ranged from 0.74–1.02.

#### Dilution integrity

To dilute patient samples above ULOQ, blank human plasma and physiological saline solution were adequate and did not affect accuracy and precision (ESM Table S2).

#### Accuracy and (im)precision

Intra-day accuracy and precision (expressed as CV in %) met the acceptance criteria (Table 1). Between-day imprecision was also found adequate (Table 1).

**Table 1:** Intra-day accuracy and precision (CV%), *n* = 5 per Level and inter-day imprecision *n* = 3

Analyte	QC level	Intra-day accuracy and precision		Inter-day imprecision		
		Accuracy (%)	CV (%)	Within-day imprecision	Between-day imprecision	Total imprecision
				CV (%)	CV (%)	CV (%)
AFA	QC-H	101.0 ± 4.20	4.18	5.14	0.00^a^	5.14
	QC-M	99.9 ± 1.47	1.48	4.02	0.00^a^	4.02
	QC-L	92.9 ± 1.02	1.10	3.76	9.87	10.6
	QC-LLOQ	99.2 ± 2.95	2.97	4.52	4.46	6.35
AXI	QC-H	93.6 ± 4.75	5.07	3.30	3.67	4.94
	QC-M	102.0 ± 5.43	5.30	4.04	0.00^a^	4.04
	QC-L	100.0 ± 3.27	3.26	2.74	0.00^a^	2.74
	QC-LLOQ	99.3 ± 4.77	4.81	5.41	4.12	6.80
BOS	QC-H	93.3 ± 7.16	7.68	5.06	0.00^a^	5.06
	QC-M	94.0 ± 2.52	2.68	3.41	0.52	3.45
	QC-L	90.0 ± 2.81	3.13	4.75	0.00^a^	4.75
	QC-LLOQ	97.8 ± 3.42	3.5	3.87	0.00^a^	3.87
CAB	QC-H	100.0 ± 2.00	1.95	5.35	1.98	5.70
	QC-M	104.0 ± 0.89	0.86	4.33	4.81	6.47
	QC-L	103.0 ± 2.88	2.79	3.84	12.6	13.2
	QC-LLOQ	105.0 ± 3.52	3.36	4.74	12.0	12.9
DAB	QC-H	102.0 ± 4.73	4.65	4.11	4.64	6.20
	QC-M	100.0 ± 3.17	3.17	3.29	3.26	4.63
	QC-L	96.1 ± 5.41	5.63	4.60	7.17	8.52
	QC-LLOQ	102.0 ± 5.71	5.59	5.23	10.4	11.6
LEN	QC-H	98.6 ± 5.96	6.04	4.11	4.64	6.20
	QC-M	99.2 ± 6.55	6.60	3.29	3.26	4.63
	QC-L	99.5 ± 1.21	1.21	2.73	1.35	3.04
	QC-LLOQ	101.0 ± 5.52	5.47	4.52	4.65	6.48
NIL	QC-H	100.2 ± 5.65	5.64	3.60	2.59	4.43
	QC-M	106.0 ± 5.94	5.63	4.02	3.48	5.32
	QC-L	102.0 ± 2.32	2.28	3.45	0.00^a^	3.45
	QC-LLOQ	106.0 ± 5.52	5.19	4.40	2.44	5.03
OSI	QC-H	94.3 ± 2.43	2.57	2.57	4.38	5.08
	QC-M	100.0 ± 3.90	3.89	3.08	6.21	6.93
	QC-L	101.0 ± 4.66	4.62	3.82	8.83	9.62
	QC-LLOQ	99.3 ± 3.93	3.96	2.83	8.30	8.77
RUX	QC-H	98.1 ± 3.58	3.65	4.37	3.04	5.32
	QC-M	102.0 ± 2.82	2.76	3.19	1.73	3.63
	QC-L	93.2 ± 2.45	2.63	3.23	2.17	3.89
	QC-LLOQ	91.7 ± 3.36	3.66	3.25	3.20	4.56
TRA	QC-H	90.2 ± 7.34	8.15	2.86	0.00^a^	2.86
	QC-M	92.3 ± 4.93	5.35	3.02	0.00^a^	3.02
	QC-L	89.03 ± 4.82	5.42	1.88	5.54	5.85
	QC-LLOQ	92.6 ± 5.82	6.28	7.80	5.97	9.82

^a^Between-day imprecision was set to zero, because variance of day averages was less than weighted variance of pooled within-day imprecision, according to Krouwer and Rabinowitz

#### Stability

Pre-preparative stability (bench-top stability) investigations of AXI, BOS, CAB, DAB, LEN, NIL, RUX, and TRA showed that they can be stored in the fridge (4 °C) for 6 weeks and for 24 h at room temperature (Table 2). Prolonged storage at room temperature and light exposure did not show any impact on accuracy and precision either (Table 2). Incubation at 56 °C for 1 h also did not affect accuracy and precision (Table 2). Post-preparative stability values (autosampler stability) were within acceptance criteria (Table 2).

**Table 2:** Validation results for bench-top and post-preparative stability (*n* = 3)

Analyte	QC level	4 °C 6 weeks		Ambient temperature without daylight (24 h)		Ambient temperature with daylight (48 h)		Ambient temperature without daylight (48 h)		Incubation at 56 °C (1 h)		Autosampler (24 h)	
		Accuracy (%)	CV%	Accuracy (%)	CV%	Accuracy (%)	CV%	Accuracy (%)	CV%	Accuracy (%)	CV%	Accuracy (%)	CV%
AFA	QC-H	66.1 ± 1.38	2.08	90.2 ± 3.34	3.71	83.5 ± 0.46	0.55	81.3 ± 3.41	4.19	86.3 ± 0.99	1.15	98.1 ± 4.73	4.82
	QC-L	38.9 ± 0.42	1.08	72.8 ± 3.50	4.80	63.7 ± 5.05	7.93	63.0 ± 0.78	1.24	94.9 ± 4.31	4.55	98.0 ± 6.10	6.23
AXI	QC-H	101.6 ± 3.11	3.07	99.9 ± 0.51	0.01	89.9 ± 1.38	1.53	96.9 ± 1.93	1.99	86.8 ± 0.57	0.65	98.7 ± 3.36	3.40
	QC-L	100.0 ± 3.83	3.83	93.7 ± 2.82	3.01	90.9 ± 2.32	2.55	97.7 ± 1.68	1.72	96.9 ± 1.48	1.53	97.9 ± 3.68	3.75
BOS	QC-H	102.1 ± 4.54	0.04	102.7 ± 0.14	0.14	100.7 ± 4.66	4.62	106.7 ± 1.42	1.33	95.7 ± 0.57	0.59	98.2 ± 3.59	3.66
	QC-L	97.9 ± 3.66	0.04	91.7 ± 6.40	6.99	96.3 ± 5.13	5.33	99.7 ± 2.56	2.56	102.8 ± 6.01	5.85	99.4 ± 2.95	2.96
CAB	QC-H	101.0 ± 1.70	1.68	96.0 ± 6.50	6.77	96.3 ± 0.82	0.85	96.7 ± 0.58	0.60	91.2 ± 3.96	4.34	94.5 ± 3.33	3.52
	QC-L	99.6 ± 0.57	0.57	98.3 ± 1.09	1.11	99.8 ± 2.79	2.79	98.4 ± 0.76	0.77	99.1 ± 1.34	1.36	91.2 ± 2.13	2.33
DAB	QC-H	98.3 ± 1.28	1.30	102.5 ± 4.54	4.43	98.5 ± 2.56	2.60	99.6 ± 1.18	1.19	94.3 ± 0.71	0.75	105.2 ± 3.50	3.32
	QC-L	94.4 ± 2.57	2.72	95.3 ± 3.61	3.79	98.8 ± 2.33	2.36	100.4 ± 1.42	1.41	99.5 ± 0.78	0.78	103.9 ± 4.74	4.56
LEN	QC-H	104.6 ± 1.19	1.14	108.0 ± 5.77	5.32	103 .9 ± 1.53	1.47	104.3 ± 0.34	0.32	90.7 ± 0.49	0.55	111.6 ± 6.08	5.45
	QC-L	99.1 ± 5.73	5.78	97.2 ± 5.59	5.76	96.3 ± 1.90	0.81	98.9 ± 0.81	0.82	94.0 ± 0.00	0.00	110.3 ± 3.79	3.43
NIL	QC-H	103.1 ± 4.60	4.47	105.2 ± 5.30	5.04	102.0 ± 3.01	2.96	104.1 ± 0.59	0.57	97.9 ± 0.85	0.87	96.1 ± 3.33	3.47
	QC-L	103.3 ± 1.00	0.97	94.1 ± 3.90	4.15	99.9 ± 2.24	2.25	97.7 ± 2.48	2.54	98.7 ± 0.71	0.72	97.1 ± 6.41	6.60
OSI^a^	QC-H	90.3 ± 3.00	3.32	–	–	6.24 ± 0.04	0.58	3.51 ± 0.27	7.58	26.6 ± 0.72	2.70	92.4 ± 2.87	3.11
	QC-L	87.9 ± 0.00	0.00	–	–	n.a.^b^	n.a.^b^	n.a.^b^	n.a.^b^	n.a.^b^	n.a.^b^	98.5 ± 2.35	2.39
RUX	QC-H	102.1 ± 2.29	2.25	95.5 ± 5.38	5.63	99.3 ± 2.61	2.63	98.5 ± 0.88	0.90	92.4 ± 2.55	2.75	92.7 ± 1.53	1.65
	QC-L	100.9 ± 4.16	4.12	98.2 ± 2.07	2.11	99.3 ± 0.46	0.46	98.8 ± 1.70	1.72	97.0 ± 5.66	5.83	93.4 ± 6.47	6.93
TRA	QC-H	99.1 ± 0.44	0.45	100.2 ± 0.63	0.63	100.8 ± 2.43	2.41	100.5 ± 1.70	1.69	94.6 ± 1.56	1.64	99.2 ± 3.27	3.30
	QC-L	105 .9 ± 0.70	0.66	89.8 ± 1.64	1.82	95.3 ± 2.54	2.67	96.6 ± 2.68	2.77	98.5 ± 3.54	3.59	93.6 ± 2.21	2.36

^a^Conditions varied in the case of OSI: 4 °C for 24 h, ambient temperature w/ and w/o daylight for 24 h

^b^Below LLOQ (6 ng/mL)

Long-term stability investigations confirmed stability at − 20 °C (≥ 86.2%, CV ≤ 5.40%) and − 80 °C (≥ 96.1%, CV ≤ 5.92%) for at least 3 months. Samples in plasma were stable during three freeze-thaw cycles (≥ 96.3% ± 2.35, CV ≤ 7.79%). Stability results are illustrated in ESM Table S5.

AFA and OSI stability differed from the remaining KI. QC-H and QC-L of AFA did not meet the acceptance criteria at 4 °C for 6 weeks (QC-H 66.1% ± 1.38, CV 2.08% and QC-L 38.9% ± 0.42, CV 1.08%) and QC-L at room temperature for 24 h (72.8% ± 3.5, CV 4.80%). Consequently, storage at room temperature for 48 h confirmed the instability of AFA at room temperature without showing any light dependency (QC-H 81.3% ± 3.41–83.5% ± 0.46, CV 4.19–0.55% and QC-L 63.0% ± 0.78 to 63.7% ± 5.05, CV 1.94–7.93%). All results are shown in Table 2.

Instability of OSI under different conditions was investigated: after 24 h at room temperature, only 6.24% ± 0.04, CV 0.58% (with daylight) and 3.51% ± 0.27, CV 7.58% (without daylight), and after incubation at 56 °C for 1 h, only 26.6% ± 0.72, CV 2.70%, of the spiked analyte were detectable in QC-H. Quantitation of OSI in QC-L resulted in values below LLOQ under these conditions. In contrast, storage at 4 °C for 24 h met the accuracy and precision criteria (87.9% ± 0.00–90.3% ± 3.00, CV ≤ 3.00%). Bench-top and post-preparative stability results are shown in Table 2.

Freeze-thaw stability of OSI missed the acceptance criteria in the third cycle for QC-L (81.5% ± 2.46, CV 3.02%). Long-term stability of OSI in human plasma was demonstrated at − 80 °C (QC-H 89.5% ± 0.49, CV 0.55%, QC-L 91.9% ± 4.82, CV 5.25%) and − 20 °C (QC-H 89.8% ± 1.47, CV 1.63%, QC-L 92.4% ± 3.31, CV 3.58%) for 4 weeks (ESM Table S5).

Stock solution stability results show that all KI, but AFA, were stable in DMSO for 4 months at − 80 °C (ESM Table S6). Working solutions in methanol were stable for 4 months (ESM Table S6).

#### Incurred sample reanalysis (ISR)

AFA sample reanalysis showed no difference. OSI difference ranged from − 5.4 to + 12.3% for three samples (= 10% of all samples).

### Analysis of patient samples

AFA concentrations were analyzed in a 74-year-old Caucasian female patient receiving 20 mg/day for treatment of EGFR-mutant metastatic lung adenocarcinoma (study inclusion 16 months after initial dose). Each of the three samples was collected 4–5 h post dose (AFA taken at home) at the patient’s routine visit every 4 months (three visits in total). The average AFA serum concentration was 11.5 ng/mL ± 4.6 ng/mL (median 11.8 ng/mL, IQR 4.60 ng/mL, range 6.81–11.8 ng/mL).

**Fig. 2:**
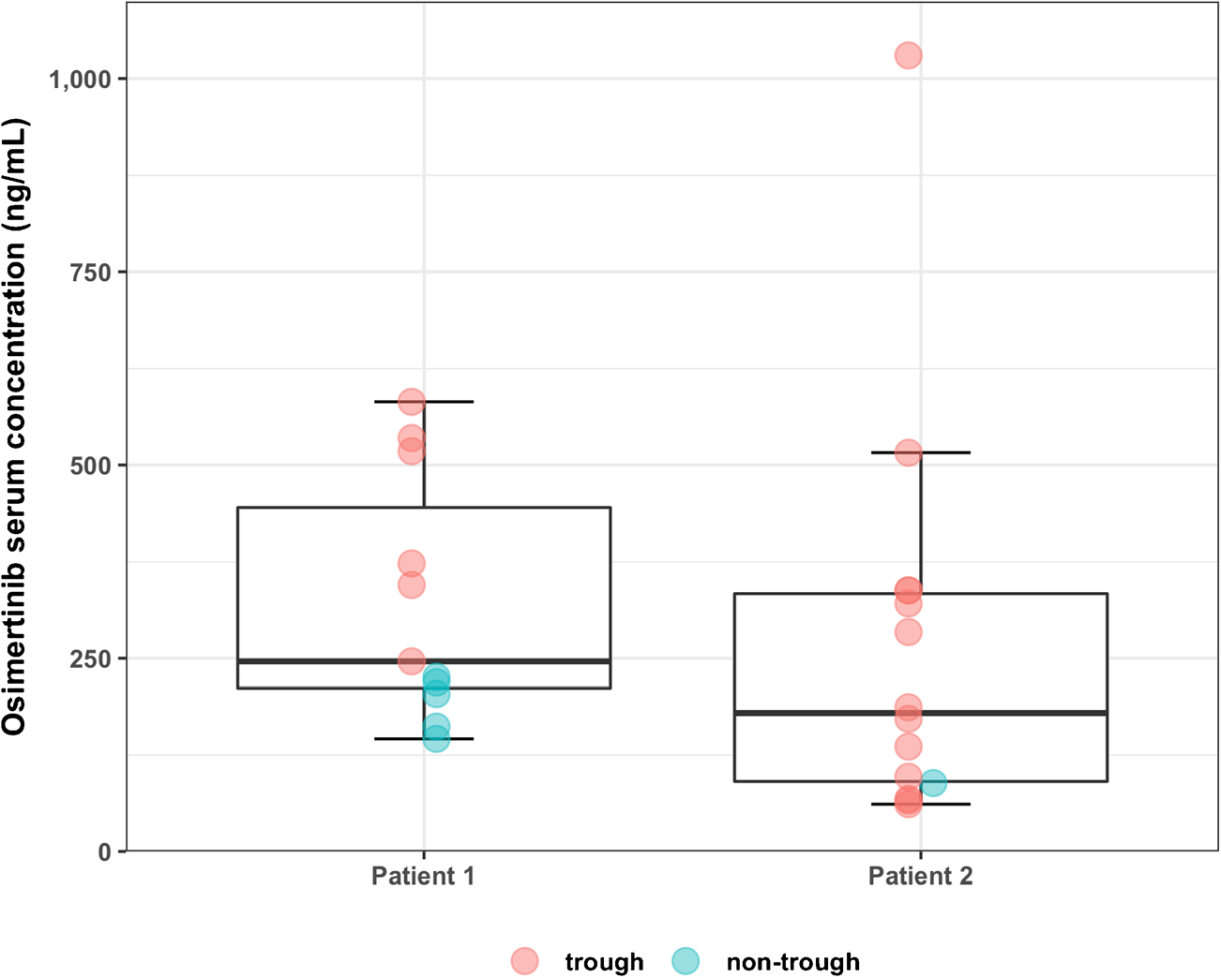
Osimertinib serum concentrations obtained from two patients treated for EGFR-mutant metastatic lung adenocarcinoma

OSI concentrations were analyzed in samples collected every 4 to 6 weeks in two Caucasian female patients (58 and 40 years, study inclusion 4 weeks after initial dose) receiving 80 mg/day OSI for treatment of EGFR-mutant metastatic lung adenocarcinoma (Fig. 2). The average OSI serum concentration of all levels in patient 1 (*n* = 11) was 323 ng/mL ± 158 ng/mL (median 246 ng/mL, IQR 234 ng/mL, range 146–582 ng/mL). The analysis of six trough levels (24.5–25 h post dose) showed an average OSI concentration of 433 ng/mL ± 131 ng/mL (median 446 ng/mL, IQR 179 ng/mL, range 246–582 ng/mL). Five samples were obtained 1–4 h post dose. Analysis of untimed OSI samples in patient 2 (*n* = 14, including 13 trough levels (23–27.5 h post dose)) revealed an average OSI serum concentration of 265 ± 260 ng/mL (median 180 ng/mL, IQR 243 ng/mL, range 61.3–1030 ng/mL).

## Discussion

Several LC-MS/MS methods for the determination of KI comprising different analyte panels (range 17 to two different KI) exist. While methods by Merienne et al. [43] Reis et al. [44], Rousset et al [45], and Nijenhuis et al. [46] use different sample preparation techniques, acidic mobile phase and C18 columns for separation, we focused on a simple and time-efficient sample preparation and used a phenyl-hexyl end-capped column at alkaline conditions. Moreover, we tried to keep the instrumentation of the LC system simple. Hence, we only used an inline filter in the autosampler and did not use any online extraction techniques or guard columns.

However, calibration ranges slightly differ in these methods: As an example, Reis et al. [44] showed a linear range from 5 to 250 ng/mL for AFA, Merienne et al. [43] calibrated AFA in a range from 4 to 800 ng/mL, whereas we found a range from 2 to 500 ng/mL to be adequate for measurements of trough levels in daily clinical setting. In comparison, our lower limit of quantification would allow determination of plasma levels prior to steady state or in PK studies.

### Sample preparation

One of the main advantages of our method is the simple and time-efficient sample preparation as it is developed to be used in daily routine. Existing methods use solid-phase extraction [43, 45], liquid-liquid extraction [46], or a mixture of both [44], which are more time-consuming. Protein precipitation resulted in high analyte recovery, a short turnaround time, and was devoid of strong matrix effects.

### Chromatographic and mass spectrometric method

Since all analytes are organic bases, weak retention and poor peak shape (excessive tailing) were the results of using formic acid, a common modifier in positive ESI, often combined with reversed phase chromatography. In contrast, a basic pH was chosen to shift the equilibrium towards the free base in order to increase analyte retention on reversed phase material. Excellent peak shape (with regard to symmetry and width) and sufficient retention were achieved using ammonium hydrogen carbonate as mobile phase additive together with a phenyl-hexyl–modified stationary phase with extended pH stability at the alkaline condition. The phenyl-hexyl modification was chosen to exploit planar π-π interactions for increased selectivity (Fig. 1). Gradient elution enabled the separation of all analytes from hydrophilic matrix compounds, while shortening retention time (elution within 3 min). In order to avoid a gradient in ionic strength, mobile phase A and B both contained ammonium hydrogen carbonate at 10 mM. Methanol was chosen as organic modifier due to the fact that ACN might undergo hydrolysis at alkaline pH (hydrolysis of nitriles to the ammonium salt of a carboxylic acid). Despite of the basic conditions, [M+H]^+^ ions were generated efficiently (comparable to using formic acid) in ESI-positive mode, due to the weakly acidic character of the hydrogen carbonate ion.

However, using an alkaline mobile phase which contains hydrogen carbonate ions impedes changing to an acidic mobile phase on the same LC system as additional washing steps are required to prevent formation of carbon dioxide in the system. In comparison to existing methods which use acetic acid [43] or formic acid [44], this can be seen as a limitation of the method’s feasibility.

Operating the column temperature at elevated temperature was not required, ensuring column stability despite high throughput and alkaline conditions, which usually tend to shorten the lifetime of chromatographic columns. Even after more than 1500 injections on the same column, retention time and analytical results remained stable.

The high analyte retention, as a result of the chosen mobile phase composition, pH, and stationary phase, enabled using the diverter valve to separate early eluting matrix components to waste, leading to effective reduction of ion suppression, ensuring reproducible and accurate analytical results. Moreover, effects of ion suppression were minimized by using isotopically labeled IS for every analyte. However, mobile phases containing ammonium hydrogen carbonate were not stable for a prolonged period of time and had to be discarded after 1 week at the latest.

During method development, the starting composition of the mobile phase for the first 0.5 min and after 5.0 min had to be adjusted to 60% because of major carryover of OSI and AXI (method development started with 40%). Carryover was assumed to be caused by OSI or AXI residues in either autosampler or MS detector valves. Hence, prior to injection, the autosampler loop was switched from inline to waste and the higher strength of the mobile phase helped removing remaining residues. Same applied to remaining residues in the MS valves. Therefore, carryover could be reduced. Furthermore, different needle wash solutions such as 90% methanol, 90% isopropanol, and a mixture of formic acid in methanol/water (0.1:50:49.9, v/v) were tested to reduce carryover. Using 90% acetonitrile minimized carryover significantly, but due to the hydrophobic character of the compounds, carryover could not be avoided completely.

Dilution integrity was tested using blank human plasma but also saline solution, since it is easily accessible in daily clinical routine. The saline solution was able to substitute blank human plasma, making it an ethically interesting alternative for this purpose.

### Recovery

To investigate recovery, samples were first prepared in water as matrix. However, solubility issues in water made it impossible to prepare matrix-free samples by serial dilution. ACN was tested as matrix instead and showed reproducible results.

### Stability

Light sensitivity of DAB in organic solvents and plasma [46] and light sensitivity of AXI [38] were reported in the literature. Investigating light exposure effects during our validation showed that DAB in plasma did not show any significant changes in concentrations, whereas concentrations of AXI in plasma decreased by about 7.0% in samples after light exposure (Table 1). While Nijenhuis et al. [46] report the instability of DAB in plasma at ambient temperature for 24 h, Herbrinks et al. [47] come to the same conclusion as we did and report stability for both compounds in plasma at room temperature for 48 h. Nijenhuis et al. concluded that DAB in plasma was only stable for a period of 6 h kept at room temperature. Unfortunately, exact conditions for their stress test (e.g., material of container and anticoagulant) were not reported impeding the direct comparison. Sparidans et al. [38] in addition confirm our findings on the stability of AXI in plasma at ambient temperature for 24 h while protected from daylight. Our method successfully separated the cis-/trans isomers of AXI [48] enabling a differentiated quantification by consideration of the photolytic degradation product.

Testing stability at 56 °C showed that at this condition, all KI, but OSI, are stable. Therefore, virus inactivation by heat (e.g., hepatitis c virus or human immunodeficiency virus and other enveloped viruses, such as SARS-CoV-2) of probably infectious or infectious samples could be performed.

Stock solution stability results show that AFA in DMSO is not stable for 4 months at − 80 °C (82.8% ± 1.23, CV 1.49%). Therefore, we recommend to either prepare stock solutions freshly or investigate further stability conditions either in other solvents or for a shorter period of time.

Stability investigations showed that AFA and OSI did not meet the acceptance criteria being kept at room temperature for more than 24 h, at 4 °C for 6 weeks and during incubation at 56 °C. These findings are in accordance with the fact that both compounds bind covalently to plasma proteins and other nucleophiles [5, 6]. The same conclusions were drawn by Veermann et al. [49] and Rood et al. [50] when they tested the stability of OSI at ambient temperature for 3 and 4 h, respectively; Veermann and colleagues suggested working on ice (+ 4 °C). As this was considered inconvenient for daily routine measurements, we chose a different approach. Working quickly through the simple sample preparation while storing and thawing samples at 4 °C produced acceptable results for accuracy and precision. However, we observed that freshly prepared calibration standards and QC samples in human matrix prior to analysis showed a better accuracy for OSI samples. Post-preparative stability investigations of OSI samples indicate that degradation and binding to matrix constituents is compensated by the IS. It is therefore crucial to use the corresponding isotope-labeled compounds for such analytes. For other analytes, CR and QC samples could be prepared in bulk and stored at − 80 °C for up to 3 months until analysis.

Rood et al. [50] reported non-linearity for OSI in their method. Having the same difficulties with assay reproducibility and accuracy, we changed the CR and QC preparation technique for OSI. Solubility issues combined with serial dilution in matrix seemed to have an effect on accuracy and precision between and within runs. Preparing the QC and CR samples differently solved the initial problems. The samples were serially diluted in organic solvent at a concentration 20 times higher compared to the intended plasma level. Each level was then individually prepared by dilution with blank matrix (1:20). Reproducibility of this assay is underlined by the ISR results.

### Patient samples

We were able to demonstrate that our assay can easily be applied in a routine clinical setting. In contrast to reported AFA levels by Wind et al. and Merienne et al. (vide infra), the analysis of three AFA samples from our patient during a year revealed low concentrations of 11.5 ng/mL ± 4.6 ng/mL, despite the fact that samples where obtained close to expected time to maximum concentration (t_max_) (4–5 h post dose). In PK investigations performed in 15 patients by Wind et al., the mean maximum concentration in steady state (C_max,ss_) at once daily oral administration of 20 mg AFA was 24.5 ng/mL (geometric CV 88.5%) [51]. Reis et al. measured 15 AFA trough concentrations in steady state (C_ss,min_) originating from an unknown number of patients and reported a mean concentration of 23.0 ± 14.4 ng/mL for a daily dose of 40 mg [44]. Merienne et al. analyzed eight trough levels in six patients receiving 40 mg/day and reported a median of 16.7 ng/mL (range 5–38.8 ng/mL) and proposed a minimum concentration (C_ss,min_) of > 10–55 ng/mL as PK target [43]. Considering that our patient was treated with 20 mg daily and given the broad range of concentrations in the above-named studies, our results seem plausible. In our patient, dose reduction from initially 40 to 30 mg and later 20 mg was performed due to severe cutaneous adverse events. On a 20 mg daily schedule, only mild adverse events remained, while the oncological situation is still stable (32 months after initiation of therapy), suggesting that lower concentrations might be necessary to control adverse events while still preventing tumor progression. This is in accordance with the findings of a retrospective study in 254 patients conducted by Lim et al. which demonstrated that a daily dose of 20 mg did not result in lower progression-free survival (PFS) in comparison to daily doses up to 40 mg, whereas significant differences in PFS could be seen when dosing regimens < 20 mg/day were applied [52].

Our results of quantification of OSI trough concentrations in clinical routine samples of 433 ng/mL ± 131 ng/mL (patient 1) and 278 ± 264 ng/mL (patient 2) are in accordance with existing data.

Rood et al. [50] reported a mean OSI trough concentration of 301.6 ± 164 ng/mL in 34 patients (2 technical replicates for each patient). Janssen et al. [53] reported a mean plasma level of 331 ng/mL (range 123–798 ng/mL) in 10 samples from an unknown number of patients. As sampling in daily clinical routine was described, we assumed that the reported concentrations also were trough levels. In contrast, Reis et al. [44] reported a slightly lower mean trough concentration of 194 ± 88 ng/mL in 15 samples obtained from an unknown number of patients. Given the broad range of trough levels in patient 2 (61–1030 ng/mL) and the significant standard deviation reported in ours, as well as other published studies, a high degree of inter- and intraindividual variability in drug exposure can be presumed, possibly promoting treatment failure or adverse events.

As both compounds are irreversible inhibitors of the EGFR, area under the curve (AUC) might be a more promising target compared to trough plasma concentrations.

## Conclusion

The developed state-of-the art LC-MS/MS method is a sensitive and rapid method for the quantification of AFA, AXI, BOS, CAB, DAB, LEN, NIL, OSI, RUX, and TRA in human serum and plasma. The application of stable isotope-labeled internal standards has proven necessary to yield acceptable stability, especially for the irreversible inhibitors AFA and OSI. We can also demonstrate that either plasma or serum can be used for the determination of drug concentrations. However, plasma should be the preferred species for the determination of osimertinib and afatinib due to the pronounced instability at ambient temperature. Samples containing these analytes should be kept at 2–8 °C immediately after sample collection and frozen as quickly as possible. Since heat inactivation of the samples was possible in the case of ruxolitinib, the method could also be used for the monitoring of patients treated with this KI for tocilizumab-refractory severe COVID-19. Our method provides the basis for further investigation on correlation between drug exposure, AE, efficiency, and other patient characteristics. Additional data from clinical routine settings need to be collected to define PK targets for an effective and safe treatment with KI.

## Electronic supplementary material

ESM 1 (PDF 449 kb)

## Data Availability

Data will be made available upon reasonable request.
