# Supplementary material for: Development and validation of a sensitive liquid chromatography tandem mass spectrometry assay for the simultaneous determination of ten kinase inhibitors in human serum and plasma
Source: Anal Bioanal Chem. 2020 Nov 6;413(2):599–612. doi: 10.1007/s00216-020-03031-7 (PMC7644392; doi:10.1007/s00216-020-03031-7)
Supplement: Supplementary file 1 — (PDF 449 kb) [file 216_2020_3031_MOESM1_ESM.pdf]

Analytical and Bioanalytical Chemistry

Electronic Supplementary Material

Development and validation of a sensitive liquid chromatography tandem mass spectrometry assay for the simultaneous determination of ten kinase inhibitors in human serum and plasma

Fatemeh Aghai, Sebastian Zimmermann, Max Kurlbaum, Pius Jung, Theo Pelzer,
Hartwig Klinker, Nora Isberner, Oliver Scherf-Clavel

Table S1 Chemical structure of the analytes. Mass transitions of analyte and internal standard were achieved by optimizing the parameters for ionization, fragmentation, and MRM-detection. Colored atoms indicate where protons/carbon-atoms are substituted by $^{2}H/^{13}C$ in the stable isotope labeled internal standards

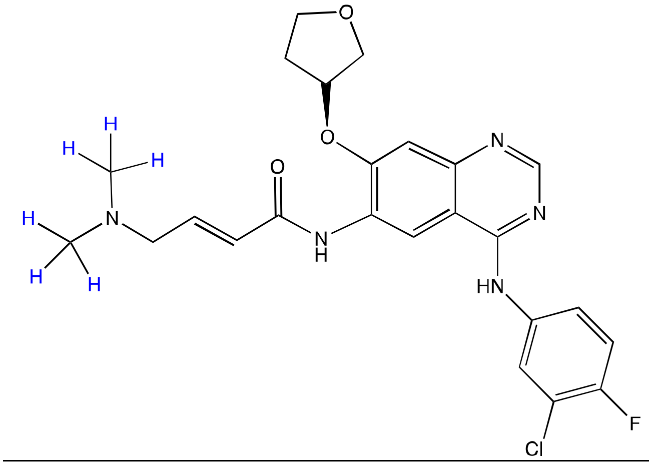	**Afatinib (AFA)**
Mass transition analyte	486.0 → 371.0
Mass transition isotopically marked internal standard	492.1 → 371.0
Retention time (R_t) (min)	3.04
Declustering potential (volts)	100.0
Enterance potential (volts)	5.0
Collision energy (volts)	34.0
Collision cell exit potential (volts)	11.0

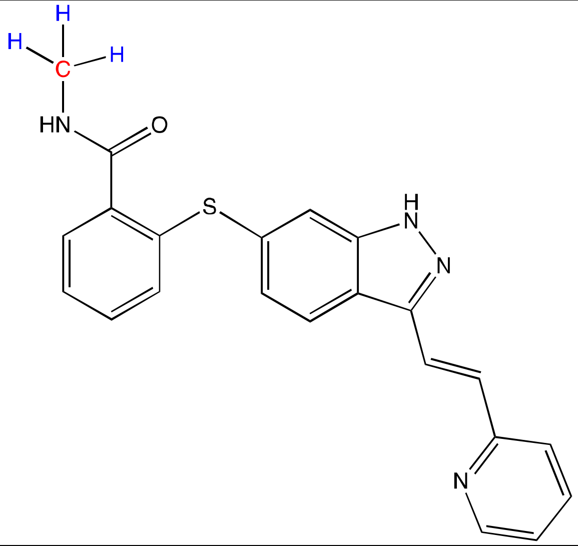	**Axitinib (AXI)**
Mass transition analyte	387.1 → 356.0
Mass transition isotopically marked internal standard	391.3 → 356.1
Retention time (R_t) (min)	1.84
Declustering potential (volts)	80.0
Enterance potential (volts)	6.0
Collision energy (volts)	27.0
Collision cell exit potential(volts)	16.0

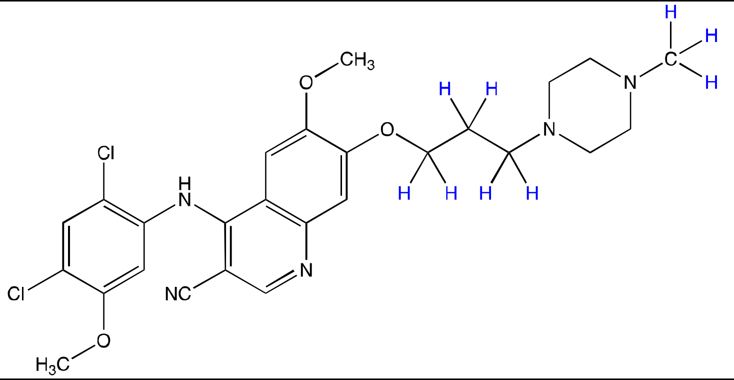	**Bosutinib (BOS)**
Mass transition analyte	530.4 → 141.3
Mass transition isotopically marked internal standard	539.4 → 150.3
Retention time (R_t) (min)	2.82
Declustering potential (volts)	80.0
Enterance potential(volts)	10.0
Collision energy (volts)	22.0
Collision cell exit potential (volts)	14.0

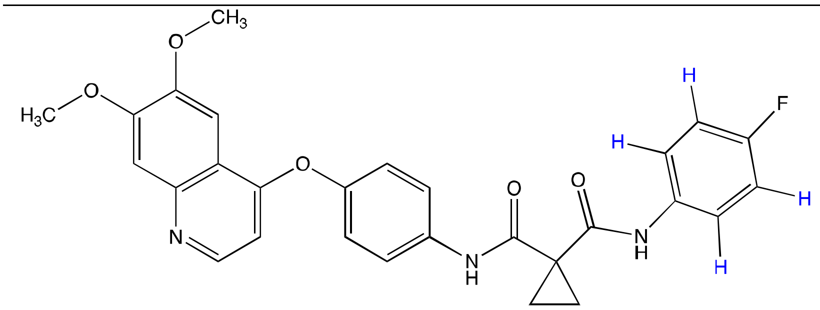	**Cabozantinib (CAB)**
Mass transition analyte	502.2 → 323.1
Mass transition isotopically marked internal standard	506.4 → 391.4
Retention time (R_t) (min)	3.42
Declustering potential (volts)	50.0
Enterance potential (volts)	10.0
Collision energy (volts)	35.0
Collision cell exit potential (volts)	14.0

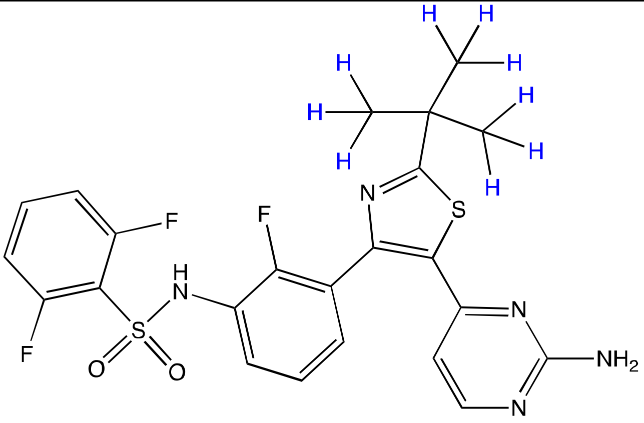

Dabrafenib (DAB)

Mass transition analyte	520.1 → 307.1
Mass transition isotopically marked internal standard	529.0 → 316.1
Retention time (R_t) (min)	1.50
Declustering potential (volts)	80.0
Enterance potential (volts)	10.0
Collision energy (volts)	30.0
Collision cell exit potential (volts)	14.0

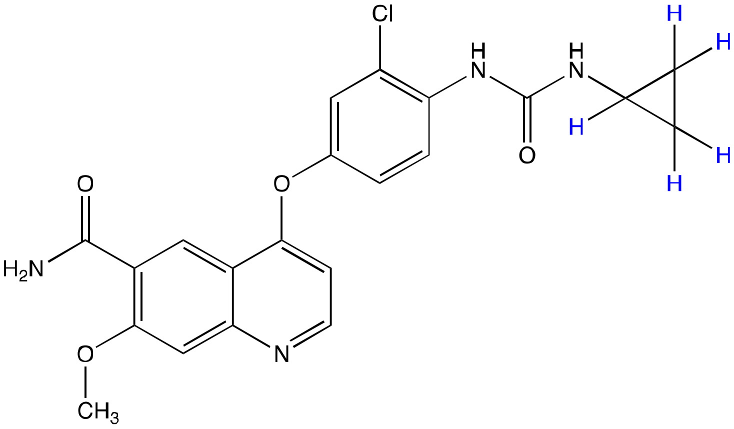

Lenvatinib (LEN)

Mass transition analyte	428.1 → 371.0
Mass transition isotopically marked internal standard	433.1 → 371.0
Retention time (R_t) (min)	1.55
Declustering potential (volts)	50.0
Enterance potential (volts)	10.0
Collision energy (volts)	43.0
Collision cell exit potential (volts)	14.0

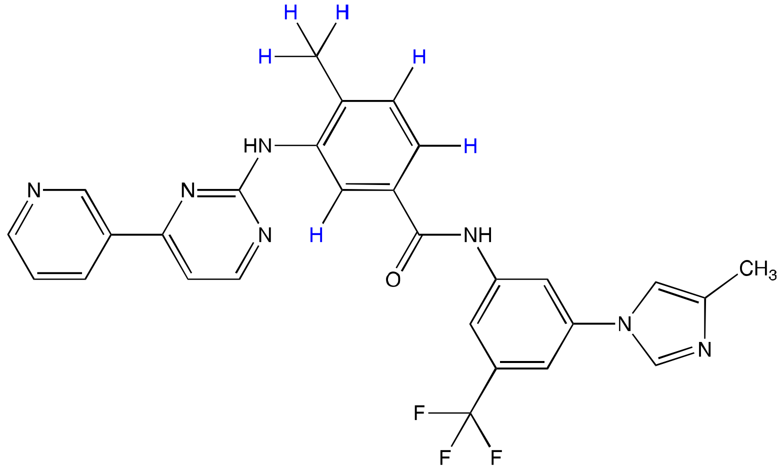	**Nilotinib (NIL)**
Mass transition analyte	530.0 → 289.1
Mass transition isotopically marked internal standard	536.1 → 295.0
Retention time (R_t) (min)	3.30
Declustering potential (volts)	100.0
Enterance potential (volts)	10.0
Collision energy (volts)	30.0
Collision cell exit potential (volts)	14.0

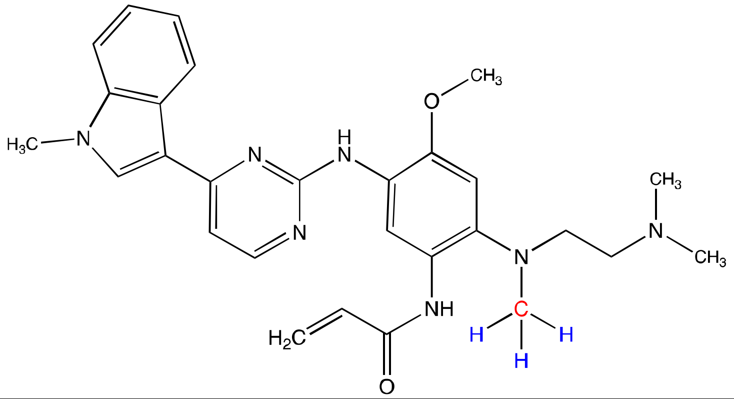	**Osimertinib (OSI)**
Mass transition analyte	500.2 → 72.1
Mass transition isotopically marked internal standard	504.2 → 72.0
Retention time (R_t) (min)	3.81
Declustering potential (volts)	50.0
Enterance potential (volts)	10.0
Collision energy (volts)	47.0
Collision cell exit potential (volts)	14.0

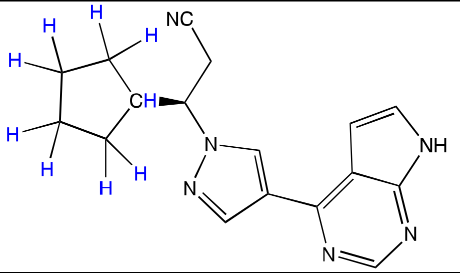	**Ruxolitinib (RUX)**
Mass transition analyte	307.0 → 186.0
Mass transition isotopically marked internal standard	316.0 → 186.0
Retention time (R_t) (min)	1.24
Declustering potential (volts)	80.0
Enterance potential (volts)	10.0
Collision energy (volts)	30.0
Collision cell exit potential (volts)	14.0

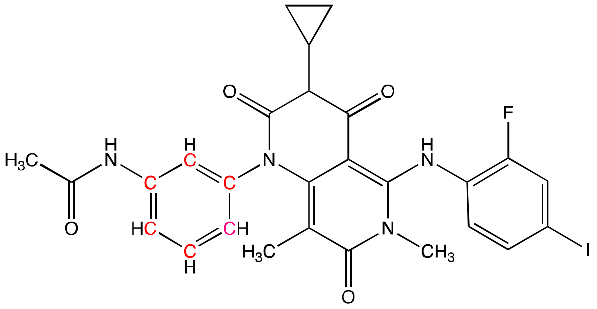	**Trametinib (TRA)**
Mass transition analyte	616.0 → 490.9
Mass transition isotopically marked internal standard	622.0 → 496.9
Retention time (R_t) (min)	3.89
Declustering potential (volts)	125.0
Enterance potential (volts)	7.0
Collision energy (volts)	46.0
Collision cell exit potential (volts)	12.0

Table S2 Validation results for sensitivity, selectivity, carry-over and dilution integrity (with saline solution)

Analyte	*Sensitivity (n=9)*	*S/N-ratio[b] (n=9)*	*Selectivity (n=6) (analyte)*	*Selectivity (n=6) (IS)*	*Carry-over (n = 5) (analyte)*	*Carry-over (n = 5) (IS)*	*Dilution integrity (n = 6)*	
	Acceptance Factor[a]	-[b]	(%)	(%)	*(%)*	*(%)*	*Accuracy (%)*	*CV%*
AFA	0.25 ± 0.20 (0.05-0.84)	20.7 ± 5.7	1.75	0.19	6.83	0.05	102.0 ± 1.33	1.39
AXI	0.13 ± 0.07 (0.01-0.31)	77.0 ± 32.6	0.15	0.01	1.84	0.01	88.4 ± 4.02	3.70
BOS	0.35 ± 0.24 (0.09-0.91)	11.9 ± 4.4	3.52	0.05	3.36	0.02	95.0 ± 2.77	2.82
CAB	0.14 ± 0.10 (0.02-0.34)	51.6 ± 18.7	2.79	0.11	4.06	0.08	89.9 ± 2.21	2.20
DAB	0.06 ± 0.03 (0.02-0.14)	34.9 ± 12.6	0.65	0.08	0.48	0.01	94.1 ± 2.64	2.80
LEN	0.02 ± 0.02 (0.01-0.08)	46.5 ±14.0	0.23	0.03	0.84	0.02	100.0 ± 2.15	2.39
NIL	0.20 ± 0.18 (0.02-0.86)	47.5 ± 19.1	2.60	0.23	7.33	0.05	98.0 ± 3.35	3.53
OSI	0.03 ± 0.01 (0.01-0.06)	25.2 ± 10.3	1.35	0.26	12.3	0.16	109.0 ± 4.31	4.87
RUX	0.03 ± 0.02 (0.01-0.11)	79.4 ± 33.5	0.28	0.30	0.71	0.36	109.0 ± 2.36	2.32
TRA	0.04 ± 0.04 (0.02-0.16)	47.0 ± 18.9	0.31	0.004	0.94	0.01	95.4 ± 1.91	1.75

[a] mean ± SD (range)

[b] S/N-ratios calculated for the lowest calibration level expressed as mean ± SD

Table S3 Accuracy and precision of quality control (QC) samples prepared in hemolytic, icteric and lipemic serum, quantified against a calibration curve prepared in plasma (n = 2)

**Analyte**	**Sample**	**Accuracy (%)**	**CV (%)**	**Accuracy (%)**	**CV (%)**	**Accuracy (%)**	**CV (%)**
		Hemolytic		Icteric		Lipemic	
AFA	QC high	96.1 ± 4.90	5.10	102.3 ± 7.36	7.19	101.2 ± 0.95	0.94
	QC low	99.4 ± 0.84	0.84	92.8 ± 3.35	3.61	92.3 ± 6.78	7.35
AXI	QC high	88.1 ± 0.44	0.50	96.3 ± 0.59	0.61	93.7 ± 0.15	0.16
	QC low	96.5 ± 1.71	1.77	96.6 ± 1.00	1.04	92.1± 8.12	8.81
BOS	QC high	95.9 ± 4.17	4.35	99.6 ± 1.34	1.35	99.8 ± 0.45	0.45
	QC low	89.4± 6.5	7.28	90.5 ± 5.08	5.61	88.6 ± 7.62	8.60
CAB	QC high	90.2 ± 2.42	2.67	96 ± 2.91	3.03	92.6 ± 2.90	3.14
	QC low	96.6 ± 0.99	1.03	91.4 ± 0.99	1.09	92.1 ± 2.65	2.87
DAB	QC high	100.2 ± 1.49	1.49	103 ± 0.49	0.48	104 ± 2.98	2.87
	QC low	105.2 ± 6.44	6.12	100.2 ± 2.03	2.03	102.1± 2.71	2.66
LEN	QC high	103.9 ± 8.89	8.56	110 ± 3.95	3.59	110.5 ± 1.27	1.15
	QC low	104.8 ± 5.77	5.51	96 ± 5.87	6.11	94.6 ± 0.96	1.02
NIL	QC high	95.6 ± 0.99	1.03	103 ± 2.47	2.40	102.2 ± 0.49	0.48
	QC low	96.3 ± 1.35	1.41	94.8 ± 2.71	2.86	94.8 ± 2.03	2.14
OSI	QC high	99.2 ± 0.00	0.00	97.5 ± 1.18	1.21	109.6 ± 5.30	4.84
	QC low	100.6 ± 0.82	0.82	101.5 ± 1.23	1.22	96.5 ± 1.64	1.70
RUX	QC high	92.2 ± 3.72	4.03	91.6 ± 3.12	3.41	93.8 ± 2.08	2.22
	QC low	99.1± 3.24	3.27	95 ± 7.90	8.32	96.8 ± 2.74	2.82
TRA	QC high	95.1± 1.30	1.36	100 ± 0.14	0.14	98.2 ± 1.30	1.32
	QC low	94.2 ± 3.05	3.24	89.3 ± 1.57	1.76	88.4 ± 1.67	1.89

Table S4 Accuracy and precision of quality control (QC) samples prepared in serum, quantified against a calibration curve prepared in plasma (n = 3)

Analyte	Sample	Accuracy (%)	CV (%)
AFA	QC high	100.4 ± 2.33	2.32
	QC low	99.3 ± 0.76	0.77
AXI	QC high	96.5 ± 2.18	2.26
	QC low	97.7 ± 1.29	1.32
BOS	QC high	104.7 ± 3.06	2.92
	QC low	97.9 ± 3.91	3.99
CAB	QC high	96.2 ± 2.75	2.86
	QC low	99.8 ± 1.95	1.95
DAB	QC high	101.9 ± 3.70	3.63
	QC low	100.4 ± 4.37	4.35
LEN	QC high	98.4 ± 2.21	2.25
	QC low	92.3 ± 0.61	0.66
NIL	QC high	101.9 ± 3.10	3.04
	QC low	98.2 ± 5.10	5.20
OSI	QC high	103.0 ± 3.00	2.91
	QC low	101.0 ± 0.45	0.45
RUX	QC high	98.8 ± 2.02	2.04
	QC low	97.0 ± 6.02	6.21
TRA	QC high	103.2 ± 4.31	4.18
	QC low	97.33 ± 2.93	3.01

Table S5 Validation results for freeze-thaw stability cycles and long-term stability (n = 3)

Analyte	QC level	*Freeze-thaw cycle 1*		*Freeze-thaw cycle 2*		*Freeze-thaw cycle 3*		*Long-term stability –20 °C (3 months)*		*Long-term stability –80 °C (3 months)*	
		Accuracy (%)	CV%	Accuracy (%)	CV%	Accuracy (%)	CV%	Accuracy (%)	CV%	Accuracy (%)	CV%
AFA	QC-H	106.6 ± 3.98	3.73	106.2 ± 3.28	3.09	105.9 ± 2.26	2.14	86.2 ± 4.66	5.40	102.1 ± 1.83	1.79
	QC-L	107.9 ± 6.57	6.09	108.5 ± 6.11	5.63	111.7 ± 3.32	2.97	103.4 ± 0.00	0.00	107.8 ± 4.57	4.24
AXI	QC-H	95.6 ± 1.94	2.02	95.9 ± 1.47	1.53	102.6 ± 1.68	1.64	91.3 ± 4.68	5.13	99.4 ± 0.70	0.71
	QC-L	97.4 ± 1.45	0.98	102.3 ± 1.45	1.41	97.6 ± 1.12	1.15	104.8 ± 0.13	0.12	105.0 ± 5.72	5.45
BOS	QC-H	105.4 ± 1.73	1.64	103.9 ± 3.22	3.10	106.3 ± 0.79	0.75	96.3 ± 8.26	8.58	104.7 ± 3.94	3.77
	QC-L	101.2 ± 2.04	2.01	99.4 ± 0.74	0.74	101.2 ± 5.04	4.98	101.8 ± 2.34	2.30	105.0 ± 6.21	5.92
CAB	QC-H	98.2 ± 4.50	4.58	98.5 ± 2.05	2.09	99.6 ± 2.16	2.17	89.7 ± 4.57	5.10	97.9 ± 2.02	2.07
	QC-L	103.5 ± 1.48	1.43	102.8 ± 2.89	2.81	105.6 ± 1.96	1.86	103.7 ± 1.27	1.22	102.0 ± 3.70	3.63
DAB	QC-H	98.1 ± 2.47	2.52	98.4 ± 1.26	1.28	100.3 ± 3.33	3.32	92.4 ± 8.79	9.51	100.1 ± 0.95	0.95
	QC-L	98.5 ± 6.74	6.84	101.5 ± 2.70	2.66	102.8 ± 0.37	0.36	99.0 ± 3.03	3.06	104.3 ± 2.97	2.85
LEN	QC-H	105.6 ± 0.63	0.59	106.0 ± 2.55	2.41	107.5 ± 3.02	2.81	92.5 ± 3.99	4.32	100.5 ± 2.44	2.43
	QC-L	103.8 ± 4.83	4.65	109.0 ± 6.64	6.09	105.3 ± 2.77	2.64	98.4 ± 1.48	1.50	97.8 ± 0.80	0.82
NIL	QC-H	97.0 ± 7.29	7.52	100.9 ± 2.94	2.91	100.6 ± 4.21	4.18	92.3 ± 5.27	5.71	98.9 ± 1.65	1.67
	QC-L	97.5 ± 6.31	6.48	98.3 ± 4.01	4.08	102.3 ± 5.93	5.79	99.4 ± 4.76	4.79	98.6 ± 2.45	2.49
OSI[a]	QC-H	93.5 ±1.47	1.57	92.8 ± 2.97	3.20	89.5 ± 1.02	1.14	89.8 ±1.47	1.63	89.5 ± 0.49	0.55
	QC-L	91.8 ±1.49	1.63	90.5 ± 2.36	2.61	81.5 ± 2.46	3.02	92.4 ± 3.31	3.58	91.9 ± 4.82	5.25
RUX	QC-H	98.6 ± 2.02	2.05	96.3 ± 7.12	7.40	98.4 ± 2.35	2.39	92.3 ± 6.13	6.64	96.1 ± 0.50	0.52
	QC-L	98.6 ± 4.65	4.72	102.5 ± 1.76	1.72	103.3 ± 2.38	2.31	95.8 ± 1.04	1.08	101.8 ± 1.40	1.38
TRA	QC-H	100.4 ± 0.50	0.50	103.7 ± 0.28	0.89	100.9 ± 1.33	1.32	93.6 ± 9.69	10.3	100.5 ± 1.00	0.99
	QC-L	100.8 ± 3.70	3.67	96.9 ± 1.47	5.72	101.5 ± 0.82	0.81	102.6 ± 7.16	6.98	101.1 ± 4.98	4.93

[a] long term stability was only assessed for four weeks in the case of OSI: - 20 °C and -80 °C

Table S6 Validation results for stock solution (SL) in DMSO and working solution in methanol (WSL) stability

Analyte	*WSL in methanol for 4 months*		*SL in DMSO for 4 months*	
	Accuracy [%]	CV% (n=3)	Accuracy [%]	CV% (n=3)
AFA	87.6 ± 0.51	0.58	82.8 ± 1.23	1.49
BOS	102.2 ± 3.04	2.97	99.2 ± 1.25	1.26
CAB	95.1 ± 3.78	3.97	89.2 ± 2.89	3.24
DAB	92.0 ± 2.89	3.14	88.2 ± 4.33	4.91
LEN	108.1 ± 2.93	2.71	109.0 ± 3.47	3.18
NIL	96.8 ± 5.09	5.26	99.6 ± 2.40	2.41
OSI	109.4 ± 2.45	2.24	105.3 ± 5.05	4.80
RUX	93.8 ± 5.03	5.36	95.0 ± 3.82	4.02
TRA	100.0 ± 2.71	2.71	103.2 ± 0.85	0.82

Table S7 Co-medication and additional condition of the patients taking OSI and AFA

**OSI**	**Patient 1**	Candesartan 24 mg QD,<br>Hydrochlothiazide 12.5 mg QD<br>Crizotinib 250 mg BID (8/14)*<br>Zolendronat every 8 weeks	Arterial hypertension
	**Patient 2**	Zolendronate every 6 weeks<br>Crizotinib 250 mg BID (3/11)*<br>Zolendronate every 6 weeks<br>Enoxaparin sodium (50.000 IE/5 mL) 0.4 mL BID	No additional conditions
**AFA**	Levothyroxine 100 µg<br>Doxycycline 50 mg QD (short-time therapy)<br>no further medication documented		Condition after thyroidectomy after papillary carcinoma of the thyroid, arterial hypertension and mitral regurgitation (Grade II classified by Carpentier)

* ratio of samples (x/total) taken during the combination of the two kinase-inhibitors (osimertinib and crizotinib)

1

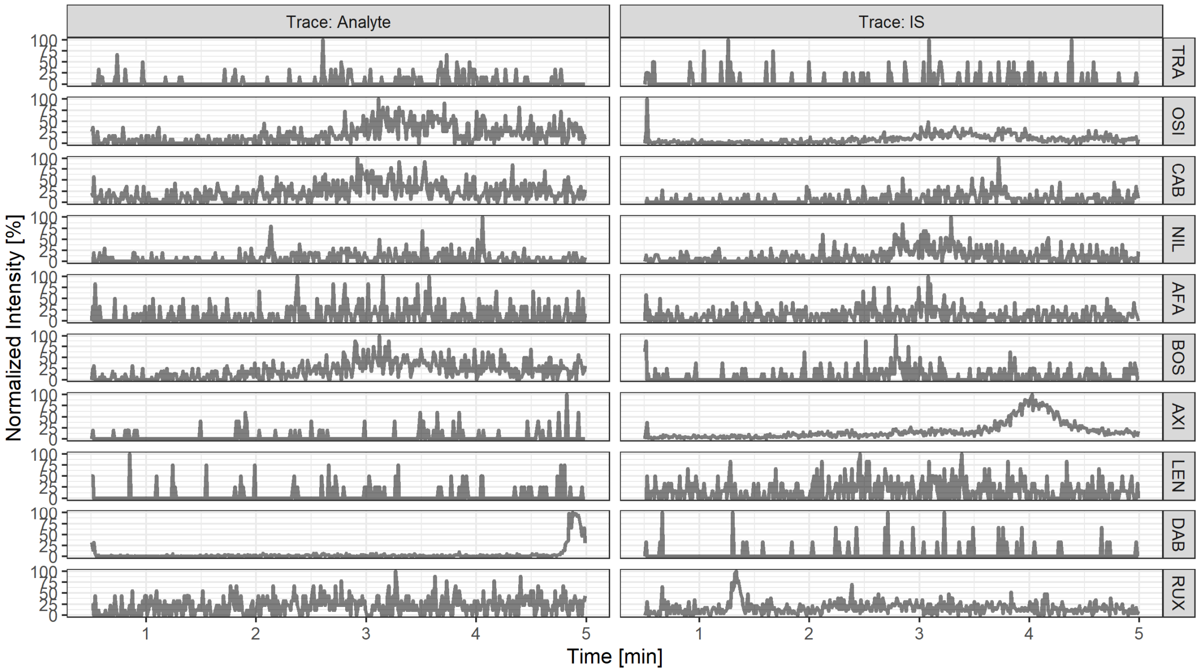

Fig. S1 Chromatographic traces of all monitored MRM transitions in a blank sample

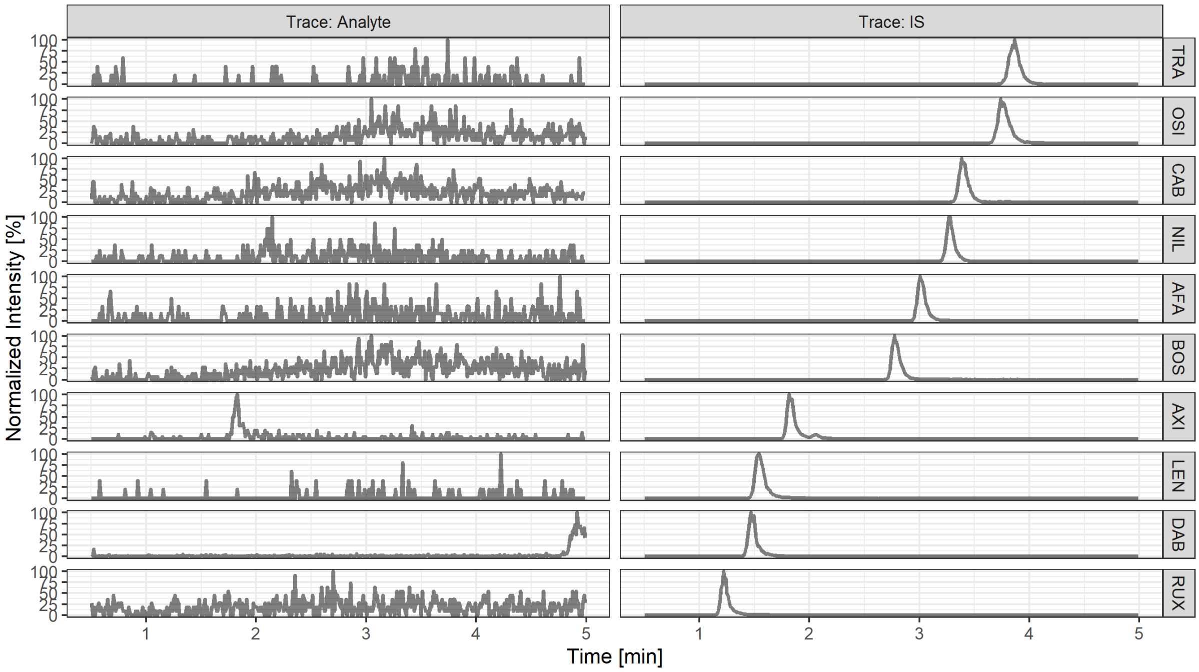

Fig. S2 Chromatographic traces of all monitored MRM transitions in a blank sample containing internal standards

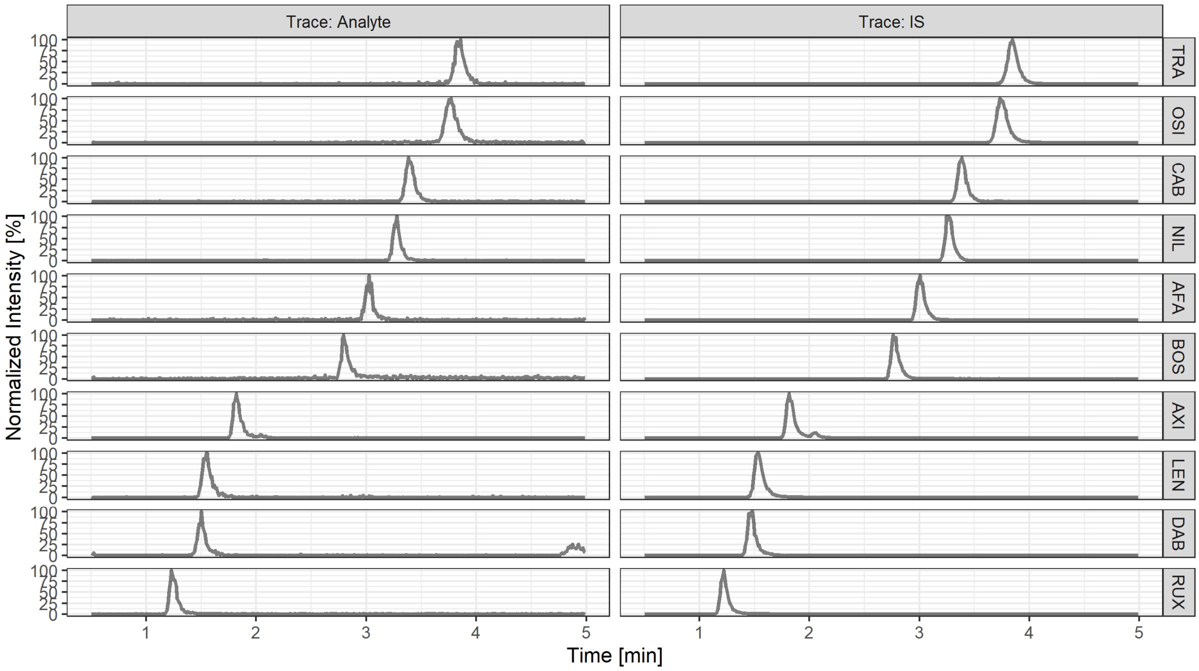

Fig. S3 Chromatographic traces of all monitored MRM transitions in an LLOQ sample
